# Supplementary material for: Predicting visual function by interpreting a neuronal wiring diagram
Source: Nature. 2024 Oct 2;634(8032):113–23. doi: 10.1038/s41586-024-07953-5 (PMC11446822; doi:10.1038/s41586-024-07953-5)
Supplement: Supplementary file 6 — CRF and ERF predictions for individual TmY4 and TmY9 cells. Analogous to Supplementary Data 3, but for TmY target types. Shown are the top four monosynaptic pathways, the strongest pathway passing through each of the top ten intermediary types (ranking from Extended Data Fig. 7), and the trisynaptic pathway Tm1–TmY–Dm3–TmY (see the section entitled Prediction of spatial normalization). [file 41586_2024_7953_MOESM6_ESM.zip › DataS4/TmY9qΓèÑ/720575940632951377.pdf]

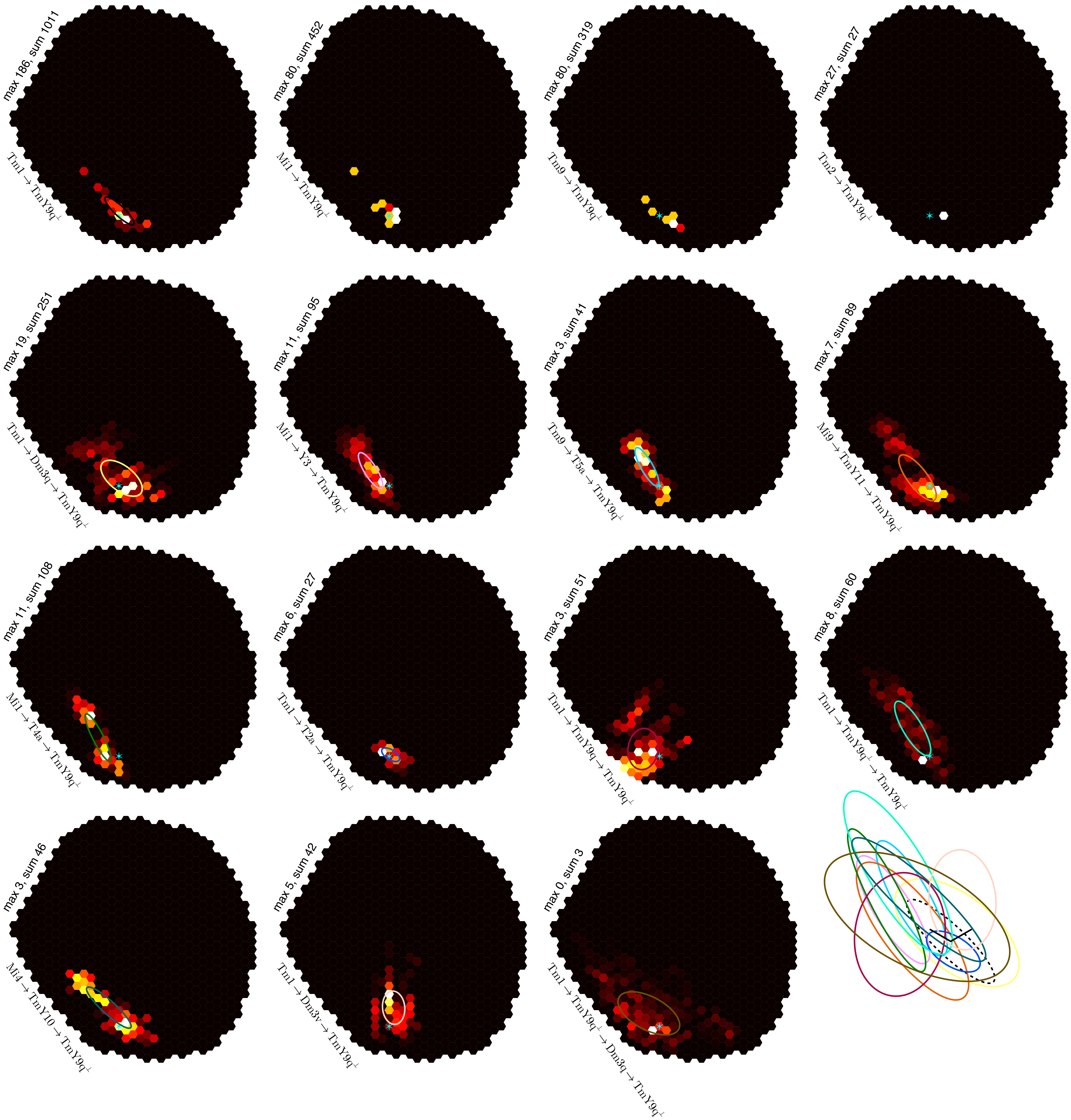
max 186, sum 1011
Tm1 → TmY9q⊥
max 80, sum 452
Mi1 → TmY9q⊥
max 80, sum 319
Tm9 → TmY9q⊥
max 27, sum 27
Tm2 → TmY9q⊥
max 19, sum 251
Tm1 → Dm3q → TmY9q⊥
max 11, sum 95
Mi1 → Y3 → TmY9q⊥
max 3, sum 41
Tm9 → T5a → TmY9q⊥
max 7, sum 89
Mi9 → TmY11 → TmY9q⊥
max 11, sum 108
Mi1 → T4a → TmY9q⊥
max 6, sum 27
Tm1 → T2a → TmY9q⊥
max 3, sum 51
Tm1 → TmY9q → TmY9q⊥
max 8, sum 60
Tm1 → TmY9q⊥ → TmY9q⊥
max 3, sum 46
Mi4 → TmY10 → TmY9q⊥
max 5, sum 42
Tm1 → Dm3v → TmY9q⊥
max 0, sum 3
Tm1 → TmY9q⊥ → Dm3q → TmY9q⊥
